# Facing the challenges of global agriculture today: what can we do about drought?

**DOI:** 10.3389/fphys.2013.00289

**Published:** 2013-10-17

**Authors:** Antonia Okono, Philippe Monneveux, Jean-Marcel Ribaut

**Affiliations:** ^1^CGIAR Generation Challenge Programme (GCP)Texcoco, Mexico; ^2^International Potato Center (CIP)Lima, Peru

**Keywords:** climate change, crop improvement, drought phenotyping, drought tolerance, experimental field design, geographic information system, molecular breeding, water management

It is estimated that the planet's demand for food and feed crops will almost double by 2050 (Foley et al., 2011). Globally, rainfed agriculture is practised in 80% of the total agricultural area and generates 62% of the world's staple food (FAOSTAT, 2011). Taking into consideration global water scarcity and increases in demand for non-agricultural uses of water, expansion of the area under irrigation in developing countries does not appear to be a realistic scenario to address the challenge of food security.

According to the latest climate change scenarios, 20-year extreme annual daily maximum temperature will likely increase by about 1–3°C by mid-21st century, and by about 2–5°C by the late 21st century, depending on the region and emissions scenario (IPCC, 2012). Based on historical data collected in Africa on more than 20,000 trials (1999–2007), each “degree day” spent above 30° reduced yield by 1% under optimal conditions, and that penalty rose up to 1.7% under water-limited conditions (Lobell et al., 2011). The impact of a changing climate is not only about temperature increase, but it is also affecting the magnitude of rainfall and its distribution, and therefore its availability at critical times of the crop cycle (Feng et al., 2013): in fact, while the total amount of rain increased in Africa over the last few years, the erratic and unpredictable nature of the drought and floods cycle also increased (Douglas et al., 2008). As such, improving the drought tolerance of crops, increasing the efficiency of water use and enhancing agricultural water productivity under rain-fed conditions is a number one priority today in a growing number of countries.

The recent genomics and bioinformatics revolutions offer real opportunities for dissecting drought tolerance into component traits, and then using genomic approaches to select plants with favorable alleles at the underlying genes. Although major achievements have been reported recently by the private sector, the development of effective systems for breeding complex traits such as drought tolerance continues to be a major challenge in the public sector, despite significant investments in research and development. Adoption of molecular breeding in developing countries remains very limited. This is due mainly to a shortage of well-trained personnel, inadequate high-throughput capacity, poor phenotyping infrastructure, and a lack of information systems or adapted analytic tools (Ribaut et al., 2010).

Created in 2003, the CGIAR Generation Challenge Programme (GCP) is a time-bound initiative ending in 2014. GCP's mission is to use plant genetic diversity, advanced genomic science and comparative biology to develop tools and technologies that will support plant breeders in the developing world in their efforts to produce better crop varieties for resource-poor farmers in drought-prone environments. Generic facilitating technologies developed by GCP include standardized phenotyping protocols, whole-plant physiology modeling, molecular breeding simulation studies, decision-support tools, procedures for creating low-cost trait diagnostics and high-throughput array-based genotyping systems. Since 2009, GCP has been coordinating the Integrated Breeding Platform (IBP). IBP is a one-stop shop where breeders can access the analytical tools and support services to manage their projects, find new knowledge and training opportunities, and access fora for discussion with peers.

Drought tolerance is the main target trait of the Programme, and genomics-assisted breeding for better crop production under water-limited conditions is at the heart of the research supported by GCP during its second phase. Good genetic studies are impossible without reliable phenotypic data, and plant phenotyping must be conducted locally. Most national breeding programmes from developing countries working in partnership in the GCP network have in common a scarcity of suitable field infrastructure for collection of accurate phenotypic data, especially for stresses such as drought. Therefore, GCP recognizes that accurate and reliable phenotyping is the main bottleneck in drought-tolerance research, and is allocating significant resources to improve crop phenotyping in target environments under different water regimes.

To achieve this objective, geographic information system (GIS) tools and soil water balance models have been used to describe the drought scenario faced by the crops in different target GCP environments, and to compare and cluster the phenotyping locations for GCP projects. Facilities and expertise in the different locations have been evaluated, needs have been prioritized, and today GCP is investing about four million US dollars to improve the local infrastructure of partners involved in GCP projects.

Complementary to the effort to improve infrastructure is the need to develop tools and protocols for improving characterization of environments and plant phenotypes, enhancing expertise in testing locations, and stimulating the development and use of innovative drought tolerance-related traits and protocols (e.g., carbon isotope discrimination, spectroradiometry, thermal imaging). This manual contributes to this effort.

The first part of this manual addresses—from a generic perspective—global issues and challenges related to environment selection and characterization, experimental field design, trait selection, and data analysis and management. The second part of the manual is crop-specific for a set of GCP target crops. Each article presents the state-of-the-art of research on drought tolerance and the protocols that are more specifically used to measure different traits for each of those crops.
